# Racial/Ethnic Disparities in the Prevalence and Trends of Autism Spectrum Disorder in US Children and Adolescents

**DOI:** 10.1001/jamanetworkopen.2021.0771

**Published:** 2021-03-05

**Authors:** Jing Yuan, Minghui Li, Z. Kevin Lu

**Affiliations:** 1 Department of Clinical Pharmacy and Pharmacy Practice, School of Pharmacy, Fudan University, Shanghai, China; 2 Department of Clinical Pharmacy and Translational Science, University of Tennessee Health Science Center, Memphis; 3 Department of Clinical Pharmacy and Outcomes Sciences, University of South Carolina, Columbia

## Abstract

This cross-sectional study uses data from the National Health Interview Survey to assess racial/ethnic disparities in the prevalence and trends of autism spectrum disorder among US children and adolescents.

## Introduction

Autism spectrum disorder (ASD) is a developmental disability characterized by repetitive behaviors and persistent impairments in social interaction and communication. The prevalence of ASD has been increasing since 2000, with inconsistent findings in racial/ethnic disparities.^1,2,3^ Over the past decade, the racial/ethnic disparities have persisted but have narrowed in response to the US Health and Human Services Action Plan to Reduce Racial and Ethnic Health Disparities.^4^ However, it remains unknown how racial/ethnic disparities have changed over time. We used recently released data from the National Health Interview Survey to assess the most recent temporal trends and racial/ethnic disparities in ASD prevalence from 2014 through 2019.

## Methods

This repeated cross-sectional study used nationally representative data for 2014 through 2019. The National Health Interview Survey uses a stratified multistage sample design and collects data on a wide range of health-related topics through in-person household interviews. The University of Tennessee Health Science Center institutional review board reviewed the study protocol and granted an exemption from full review. Informed patient consent was also waived because the study was a secondary analysis of deidentified data. We followed the Strengthening the Reporting of Observational Studies in Epidemiology (STROBE) reporting guideline for cross-sectional studies.

Race/ethnicity for this study was self-reported. Following the design and estimation guidelines for the National Health Interview Survey survey,^5^ we used a survey procedure in SAS version 9.4 (SAS Institute Inc) to account for the complex sampling design, and analyzed the data for race-specific prevalence and by sociodemographic and clinical characteristics. To identify time trends across survey years, we performed weighted linear regression analyses, in which the survey year was treated as a continuous variable, with adjustment for potential confounders. The SEs were estimated using Taylor series linearization, and 2-sided *P* < .05 indicated statistical significance.

## Results

In this nationally representative survey of US children and adolescents aged 3 to 17 years, 1330 of the 52 550 eligible individuals (2.53%) had been diagnosed with ASD between 2014 and 2019. Among those with ASD, the mean (SD) age was 10.58 (4.20) years; 1036 (77.89%) were males, 294 (22.11%) females, 689 (51.80%) non-Hispanic White individuals, 269 (20.23%) non-Hispanic Black individuals, and 240 (18.05%) Hispanic individuals. The overall weighted prevalence was 2.49% (95% CI, 2.29%-2.68%). The prevalence of ASD was 2.65% (95% CI, 2.40%-2.90%) in non-Hispanic White individuals, 2.85% (95% CI, 2.36%-3.33%) in non-Hispanic Black individuals, and 1.94% (95% CI, 1.64%-2.24%) in Hispanic individuals (Table). Across the 6-year study period, the weighted prevalence of ASD increased slightly from 2.24% (95% CI, 1.90%-2.58%) in 2014 to 2.79% (95% CI, 2.34%-3.24%) in 2019 (*P* = .32 for trend) (Figure). The ASD prevalence in non-Hispanic White individuals remained stable (2.55% [95% CI, 2.00%-3.10%] in 2014 and 2.54% [95% CI, 1.67%-3.41%] in 2019; *P* = .47 for trend). There were no statistically significant changes in other racial ethnic groups. However, the prevalence increased by 43% from 2.21% (95% CI, 1.25%-3.17%) to 3.16% (95% CI, 2.50%-3.81%) in non-Hispanic Black individuals (*P* = .07 for trend), and by 40% from 1.49% (95% CI, 1.10%-1.87%) to 2.08% (95% CI, 1.09%-3.07%) in Hispanic individuals (*P* = .08 for trend). An increasing prevalence in non-Hispanic Black individuals was observed in the younger age group (3.02; 95% CI, 2.33-3.70 among those aged 3-11 years vs 2.60; 95% CI, 1.99-3.21 among those aged 12-17 years).

**Table.** Weighted Prevalence of Autism Spectrum Disorder in US Children Aged 3 to 17 Years by Race/Ethnicity in the National Health Interview Survey, 2014-2019^a^

Characteristics	Prevalence, % (95% CI)				*P* value^b^
	Non-Hispanic White individuals	Non-Hispanic Black individuals	Hispanic individuals	Non-Hispanic other individuals	
Overall	2.65 (2.40-2.90)	2.85 (2.36-3.33)	1.94 (1.64-2.24)	2.24 (1.73-2.74)	.002
Age, y					
3-11	2.40 (2.06-2.73)	3.02 (2.33-3.70)	2.23 (1.79-2.67)	2.30 (1.68-2.91)	.13
12-17	3.00 (2.62-3.39)	2.60 (1.99-3.21)	1.49 (1.11-1.86)	2.14 (1.23-3.06)	<.001
Sex					
Male	4.11 (3.69-4.54)	4.11 (3.29-4.94)	2.73 (2.24-3.23)	3.40 (2.54-4.26)	.002
Female	1.12 (0.87-1.36)	1.53 (1.07-1.98)	1.12 (0.80-1.44)	1.05 (0.58-1.51)	.28
Highest educational level of family members^c^					
High school	3.02 (2.40-3.64)	2.51 (1.68-3.34)	1.70 (1.26-2.14)	2.65 (1.42-3.88)	.006
College	2.51 (2.19-2.83)	3.34 (2.63-4.05)	1.99 (1.58-2.41)	2.70 (1.91-3.50)	.005
Graduate school	2.69 (2.18-3.20)	1.82 (1.03-2.60)	3.27 (1.72-4.82)	1.15 (0.60-1.70)	.01
Family income to poverty ratio^d^					
<1.0	3.47 (2.51-4.44)	2.72 (1.86-3.57)	1.79 (1.20-2.38)	3.48 (2.16-4.80)	.01
1.0-2.0	3.30 (2.66-3.94)	3.50 (2.55-4.44)	2.11 (1.55-2.67)	2.89 (1.62-4.16)	.03
2.0-4.0	2.45 (2.05-2.86)	3.17 (1.94-4.39)	1.94 (1.34-2.55)	2.32 (1.27-3.38)	.25
>4.0	2.39 (1.99-2.79)	1.83 (1.02-2.65)	1.52 (0.71-2.33)	1.29 (0.65-1.93)	.051
Region					
Northeast	3.30 (2.69-3.92)	2.48 (2.01-2.94)	2.52 (2.08-2.95)	2.51 (1.98-3.04)	.001
Midwest	2.48 (2.01-2.94)	2.84 (2.03-3.64)	2.12 (1.10-3.14)	2.43 (1.30-3.57)	.73
South	2.52 (2.08-2.95)	2.48 (1.88-3.08)	1.84 (1.38-2.30)	2.84 (1.65-4.02)	.14
West	2.51 (1.98-3.04)	2.34 (1.30-3.37)	1.78 (1.29-2.27)	2.28 (1.53-3.02)	.26
Asthma	3.98 (3.11-4.84)	4.77 (3.38-6.17)	3.39 (2.38-4.39)	2.58 (1.33-3.82)	.15
Learning disability	20.24 (18.04-22.45)	18.47 (14.30-22.65)	17.10 (14.12-20.07)	22.23 (17.33-27.13)	.33
ADHD/ADD	12.20 (10.76-13.64)	11.89 (8.77-15.00)	11.94 (8.93-14.95)	13.88 (10.08-17.68)	.90

Abbreviations: ADHD, attention-deficit/hyperactivity disorder; ADD, attention deficit disorder.

^a^ Weighted point estimates were estimated using SAS version 9.4 survey procedures (SAS Institute Inc). Race/ethnicity was self-reported and categorized based on the 1997 Office of Management and Budget Standards.

^b^ *P* values were estimated for the difference in prevalence by strata.

^c^ Data for children with unknown family educational levels or family incomes were not estimated.

^d^ The family income to poverty ratio was calculated as the ratio of the family’s income to the respective federal poverty threshold.

**Figure.**
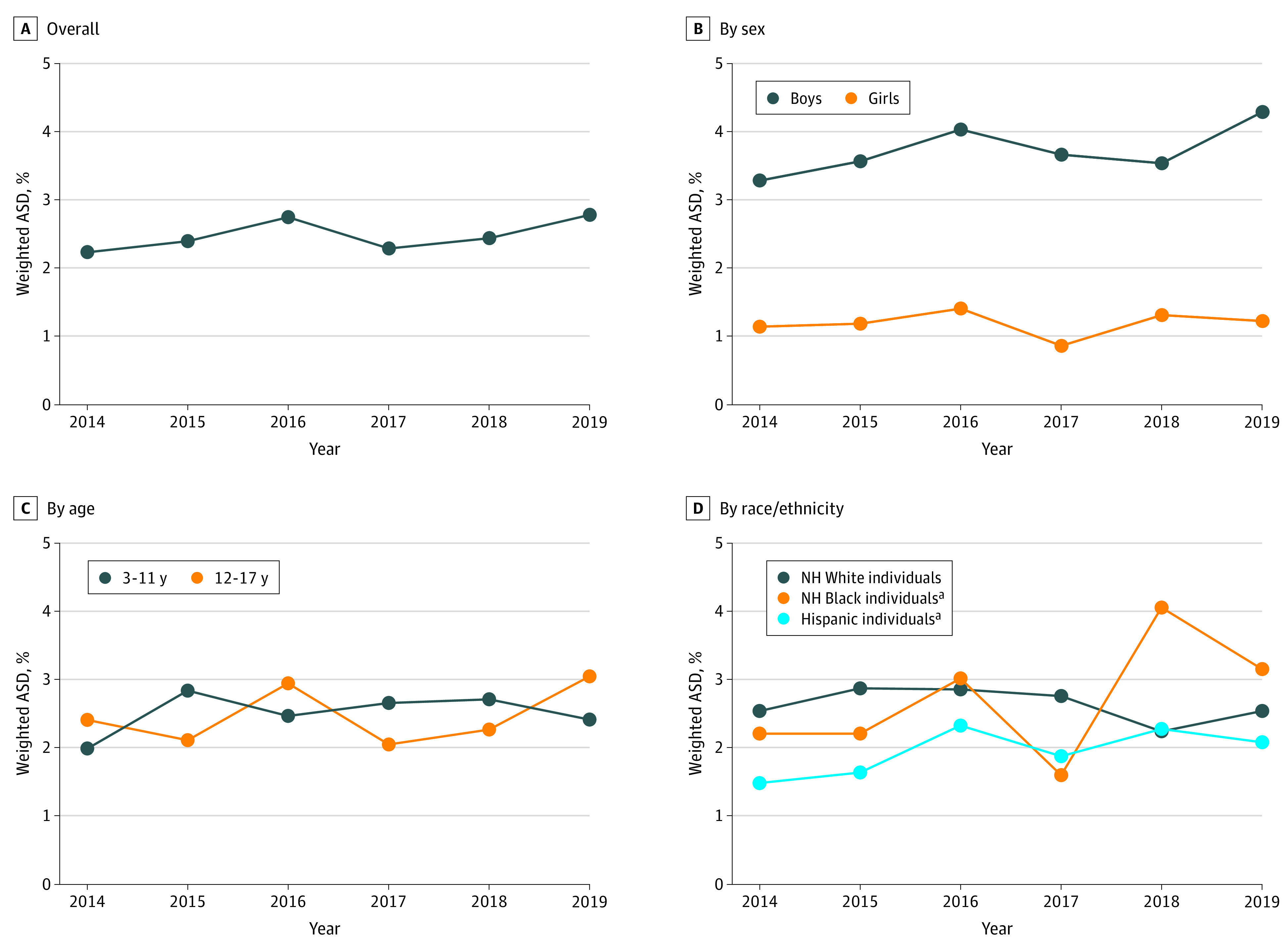
US Trends in the Prevalence of Autism Spectrum Disorder (ASD) in Children and Adolescents Aged 3 to 17 Years, 2014-2019 NH indicates non-Hispanic. ^a^*P* < .10.

## Discussion

Our findings suggest racial/ethical disparities in the temporal trend of ASD prevalence, although these differences were not statistically significant. A higher prevalence of ASD in White individuals was previously reported,^1,6^ whereas our analysis indicated that the prevalence in non-Hispanic Black individuals has surpassed that of White individuals since 2018, which is consistent with a recent study using data from the Individuals With Disabilities Education Act.^3^ More important, the increasing prevalence in Black individuals was linked to diagnosis of ASD at a younger age, potentially explained by the improved access to health care in recent years—this is the good news. However, the bad news is that because of the racial/ethnic inequities, many new cases of ASD have not been identified yet.

The decreasing trend in the prevalence of ASD between 2016 and 2018, especially the plateau in non-Hispanic White individuals, may suggest a stabilization of the environmental factors. The racial/ethnic disparities in ASD are complex and reflect multiple levels of inequities. These inequities range from individual etiologic factors (eg, genetic factors) and nonetiologic factors (eg, disease awareness and access to ASD evaluation)^5^ to environmental etiologic factors (eg, preterm birth and social experience in infancy). The main limitation of this study was the ascertainment of ASD cases, which was based on household respondents’ self-reports, rather than physicians’ evaluations. Further analysis to explore the racial/ethnic disparities by educational level, family income, and health insurance is necessary.
